# Atraumatic anterior hip dislocation after cephalomedullary nailing for an intertrochanteric femoral fracture: a case report highlighting the importance of initial reduction and prevention of excessive telescoping

**DOI:** 10.1186/s12891-026-10089-4

**Published:** 2026-06-15

**Authors:** Nobuaki Sato, Kohei Hashimoto, Yukio Nakamura, Nobunori Takahashi, Takkan Morishima

**Affiliations:** 1 Department of Orthopaedic Surgery, Tajimi City Hospital, 3-43 Maebata - cho, Tajimi, Gifu 507-8511 Japan; 2 Department of Orthopaedic Surgery, Aichi Medical University, 1-1 Yazakokarimata, Nagakute, Aichi 480-1195 Japan; 3 Division of Osteoporosis, Joint Disease Center, Department of Orthopaedic Surgery, Aichi Medical University, Locomotive Syndrome, 1-1 Yazakokarimata, Nagakute, Aichi 480-1195 Japan

**Keywords:** Intertrochanteric fracture, Anterior hip dislocation, Cephalomedullary nail, Malreduction, Telescoping, Hip instability

## Abstract

**Background:**

Anterior hip dislocation after internal fixation of a proximal femoral fracture is extremely rare. Previously reported cases have mainly involved trochanteric fractures treated with a dynamic hip screw or sliding hip screw [1–3]. To our knowledge, atraumatic anterior hip dislocation after cephalomedullary nailing for an intertrochanteric femoral fracture has not been previously reported.

**Case presentation:**

An 87-year-old woman sustained a left intertrochanteric femoral fracture (AO/OTA 31-A3) after a fall in her residential facility. Before injury, she ambulated with a walker. Her medical history included osteoporosis and vertebral compression fractures. The Beighton score was 1/9. The fracture was treated with a long TFNA nail (130°, 320 mm × 10 mm) with 2.5 cc of cement augmentation. Weight-bearing training was started on the first postoperative day, and she was transferred to another facility 1 month after surgery. Four months after surgery, she was found to have an anterior hip dislocation without any definite traumatic episode. Closed reduction under general anesthesia was unsuccessful, and conversion to total hip arthroplasty was subsequently performed 1 week later.

**Results:**

Femoral anteversion was assessed on computed tomography using the posterior condylar axis of the knee as the reference and was approximately 31°. Retrospective computed tomography review demonstrated progressive telescoping of 8.96 mm, varus collapse, and lack of medial cortical healing at the intertrochanteric fracture site. These findings suggested persistent mechanical instability at the fracture site, with secondary loss of offset and periarticular soft-tissue tension around the hip. Total hip arthroplasty was performed using the AMIS-K anterior approach. A 32-mm ceramic head on highly cross-linked polyethylene was used, and cementation was performed with Simplex bone cement (Stryker). No redislocation or other complication was observed during 1 year of follow-up.

**Conclusions:**

This case highlights not only the rarity of anterior hip dislocation after cephalomedullary nailing for an intertrochanteric fracture, but also the importance of targeting positive medial cortical support rather than purely anatomical reduction at the index surgery, and preventing excessive postoperative telescoping. Residual malreduction, insufficient medial support, and progressive shortening may result in an atypical failure pattern, including hip instability, rather than more familiar complications such as cut-out or nonunion.

## Introduction

Intertrochanteric femoral fractures are technically demanding injuries because fracture reduction is strongly influenced by the deforming muscle forces acting on each fragment. In the reverse oblique pattern, when the lesser trochanter remains attached to the distal fragment, the proximal fragment (comprising the femoral head, neck, and greater trochanter) is deformed into abduction and external rotation by the gluteal muscles and short external rotators, whereas the distal fragment is pulled into flexion, external rotation, and adduction by the iliopsoas and adductor muscles. This opposing deformity pattern makes reduction particularly challenging and may hinder the achievement of positive medial cortical support. Common complications after fixation include delayed union, nonunion, cut-out, implant failure, and malreduction [1, 2]. In contrast, hip dislocation after internal fixation of a proximal femoral fracture is rare, and anterior dislocation is particularly uncommon [3–5].

The few previously reported cases of postoperative anterior hip dislocation have mainly involved trochanteric fractures treated with a dynamic hip screw or sliding hip screw [3–5]. Proposed mechanisms have included valgus reduction, loss of horizontal offset, capsular stripping, progressive shortening, and rotational malalignment [3–9]. To our knowledge, atraumatic anterior hip dislocation after cephalomedullary nailing for an intertrochanteric femoral fracture has not been previously reported.

However, the importance of the present case lies not only in its rarity. More importantly, it illustrates that in reverse oblique intertrochanteric fractures, the quality of the initial reduction may influence not only fracture healing and implant-related complications, but also unexpected instability patterns. We therefore report this case to emphasize the importance of targeting positive medial cortical support in the index surgery, preserving medial cortical contact, and preventing excessive postoperative telescoping [1, 4, 10, 11].

## Case presentation

**Fig. 1:**
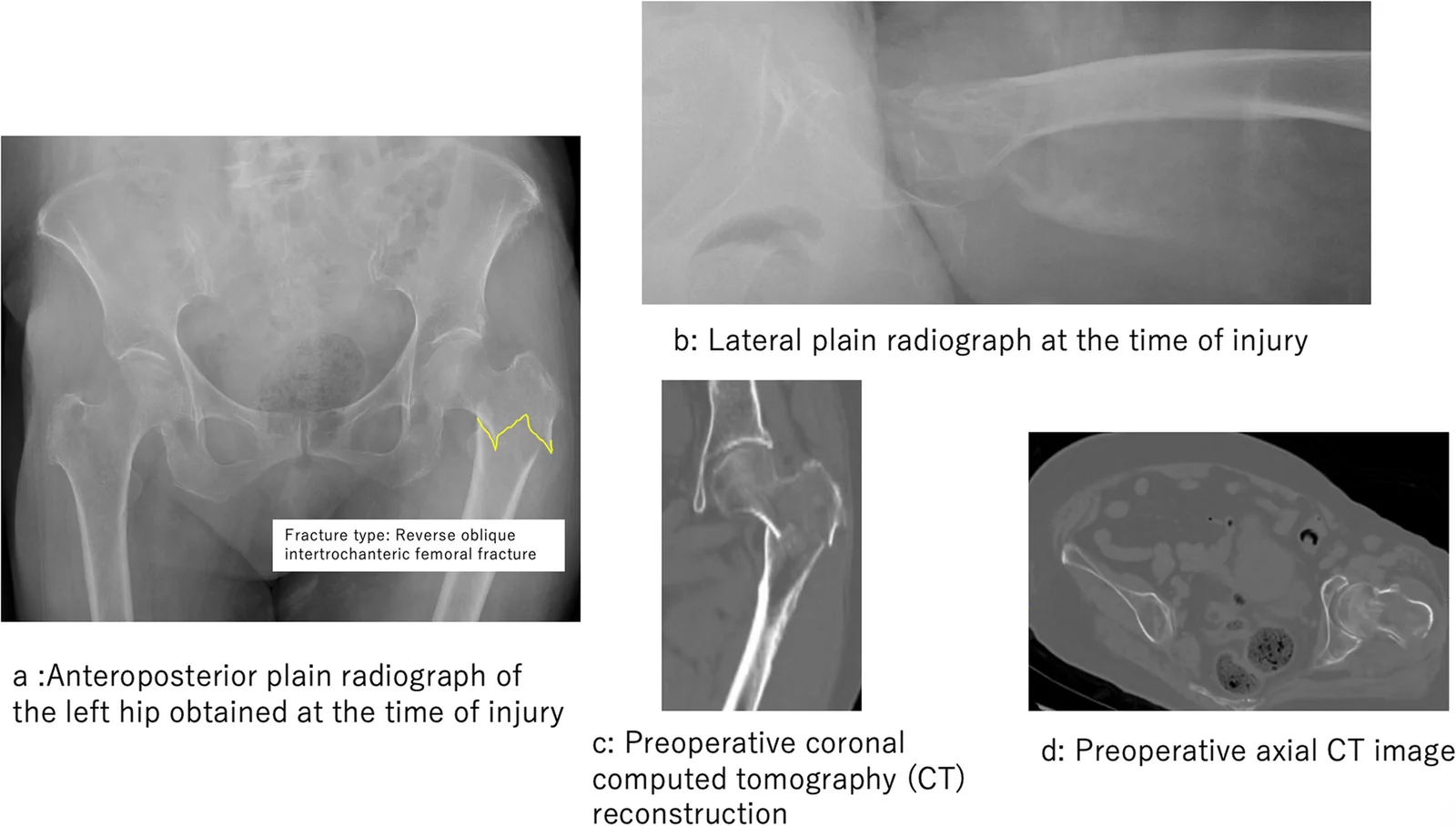
Initial injury imaging. **a** Anteroposterior plain radiograph of the left hip obtained at the time of injury, demonstrating a reverse oblique intertrochanteric femoral fracture (AO/OTA 31-A3) with disruption of the medial cortex and a separate greater trochanteric fragment. **b** Lateral plain radiograph at the time of injury, showing the sagittal configuration of the fracture. **c** Preoperative coronal computed tomography (CT) reconstruction confirming the reverse oblique fracture pattern and demonstrating limited medial cortical contact between the proximal and distal fragments. **d** Preoperative axial CT image at the level of the intertrochanteric region, illustrating the rotational orientation of the proximal and distal fragments and the position of the greater trochanteric fragment

An 87-year-old woman sustained a left intertrochanteric femoral fracture after a fall in her residential facility. The fracture was classified as AO/OTA 31-A3 (reverse oblique pattern). Before injury, she was ambulatory with a walker. Her medical history included osteoporosis and vertebral compression fractures. Generalized joint hypermobility was not supported clinically; the Beighton score was 1/9, although osteoarthritis and contractures of the elbows and knees were present (Fig. 1).

**Fig. 2:**
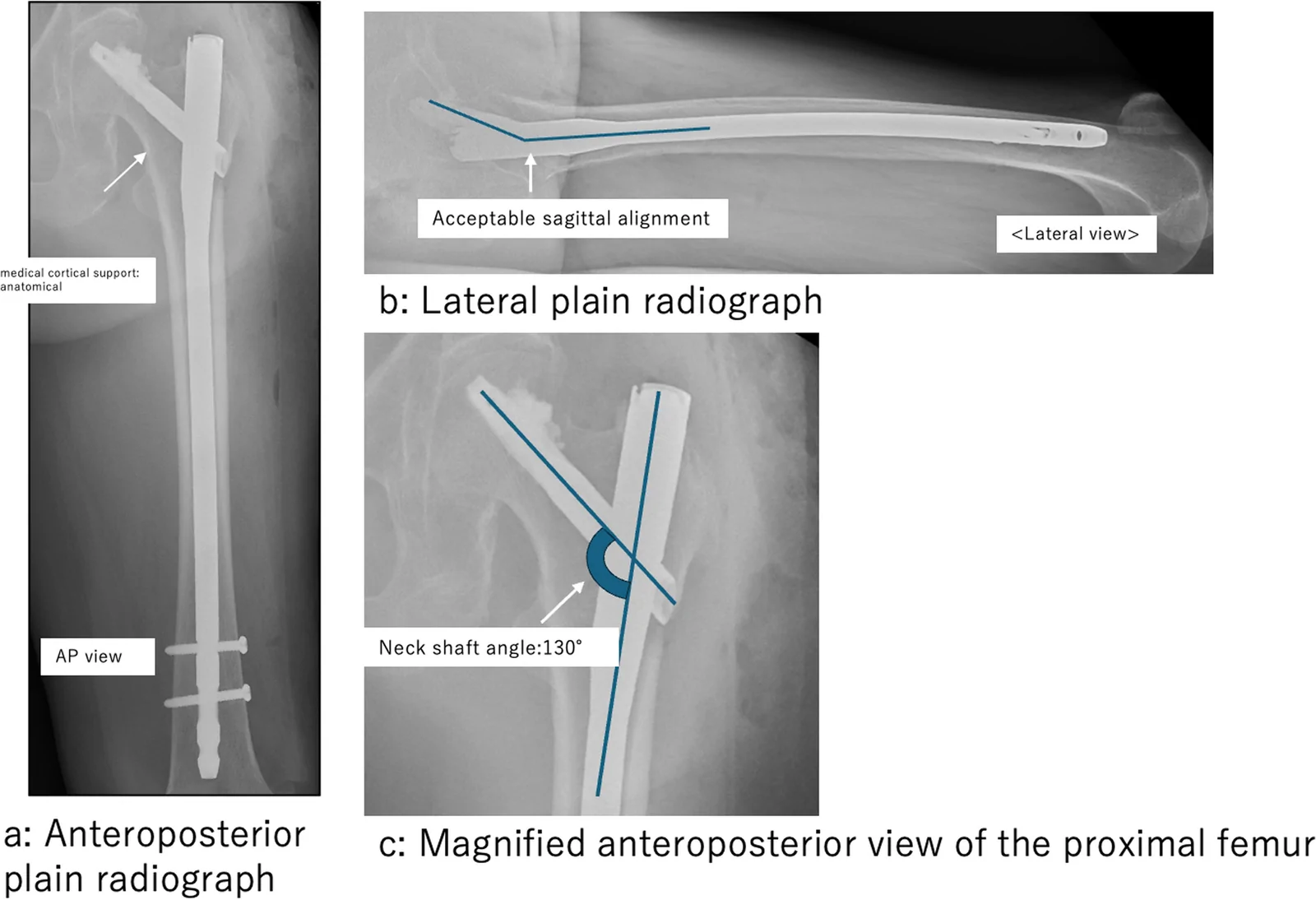
Immediate postoperative imaging after cephalomedullary nailing. **a** Anteroposterior plain radiograph obtained immediately after the index surgery (postoperative day 0), showing fixation with a long TFNA cephalomedullary nail (130°, 320 mm × 10 mm) with helical blade and cement augmentation. The neck-shaft angle measured 130° and the tip-apex distance was below 10 mm. **b** Lateral plain radiograph immediately after surgery, confirming acceptable sagittal alignment of the proximal and distal fragments and central position of the helical blade in the femoral head on the lateral view. **c** Magnified anteroposterior view of the proximal femur with annotation of the medial cortical alignment. The medial cortices of the proximal and distal fragments are aligned but without overlap, corresponding to neutral medial cortical support; positive medial cortical support was not achieved

The fracture was treated with a long TFNA cephalomedullary nail (130°, 320 mm × 10 mm) and 2.5 cc of cement augmentation. To achieve acceptable reduction, a small elevator was introduced laterally through a limited incision along the anterior surface of the intertrochanteric line to manipulate the proximal fragment [12]. Weight-bearing training was started on the first postoperative day, and the patient was transferred to another facility 1 month after surgery (Fig. 2).

**Fig. 3:**
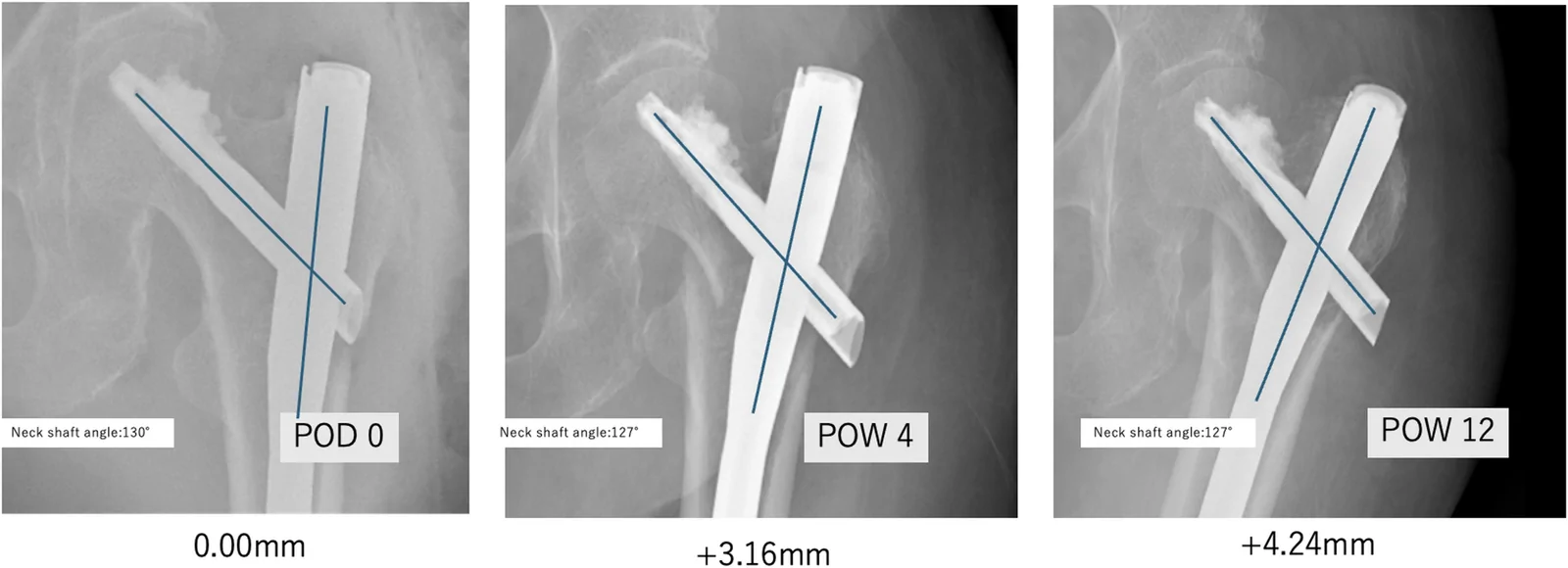
Serial postoperative anteroposterior radiographs documenting progressive telescoping. Anteroposterior plain radiographs at postoperative day 0 (left), postoperative week 4 (middle), and postoperative week 12 (right). Progressive telescoping of the cephalic element along the nail axis is evident over time, with overall sliding of approximately 8.96 mm measured between the immediate postoperative study and the time of dislocation. The neck-shaft angle was largely preserved (130°), consistent with prevention of varus collapse by cement augmentation, while axial shortening continued to progress

Femoral anteversion was assessed on computed tomography using the posterior condylar axis of the knee as the reference and was approximately 31°. The contralateral femoral anteversion was approximately 28°. Telescoping was also assessed on computed tomography by measuring the amount of blade sliding between the immediate postoperative study and the CT obtained after dislocation. Retrospective review demonstrated progressive telescoping of 8.96 mm. Computed tomography also confirmed persistent nonunion at the intertrochanteric fracture site and lack of medial cortical healing (Fig. 3; Table 1).

**Table 1:** Chronological changes in telescoping distanceand neck-shaft angle after cephalomedullary nailing

Time after surgery	Change in telescoping frompostoperative day 0 (mm)	Neck-shaft angle(° )	Change in neck-shaft angle frompostoperative day 0 (° )
Postoperative day 0	0.00	130	0
4 weeks postoperatively	+3.16	127	-3
12 weekspostoperatively	+4.24	127	-3
At dislocation	+8.96	127	-3

Telescoping distance progressively increased from 0.00 mm on postoperative day 0 to4.24mm at 12 weeks postoperatively. The neck-shaft angle decreased from 130° to 127°

**Fig. 4:**
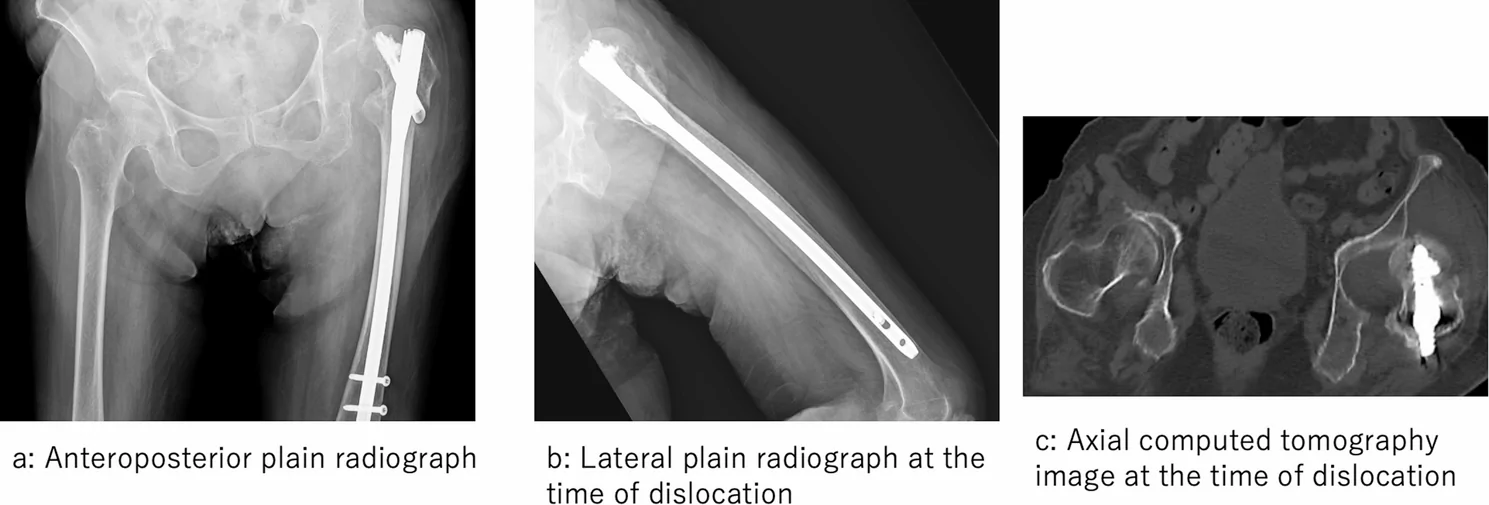
Imaging at the time of anterior hip dislocation. **a** Anteroposterior plain radiograph obtained 4 months after the index surgery, demonstrating anterior dislocation of the femoral head without any documented traumatic episode. **b** Lateral plain radiograph at the time of dislocation, confirming the anterior position of the femoral head relative to the acetabulum. **c** Axial computed tomography image at the time of dislocation showing the femoral head displaced anteriorly. No interposed bone fragment was identified between the femoral head and the acetabulum; the implant position remained unchanged from the immediate postoperative state. Femoral anteversion measured at this time was approximately 31° (contralateral side, 28°)

Four months after the index surgery, the patient was undergoing sitting rehabilitation at the facility when anterior dislocation of the left hip was identified (Fig. 4). No definite traumatic episode was documented. She was transferred back to our hospital, where closed reduction under general anesthesia and fluoroscopic guidance was attempted but was unsuccessful. Despite repeated maneuvers under full muscle relaxation, the femoral head could not be brought into the acetabulum. The implant position appeared unchanged on fluoroscopy, and no interposed bone fragment was identified between the femoral head and the acetabulum; the findings were therefore considered most consistent with soft-tissue obstruction rather than a discrete mechanical block .

**Fig. 5:**
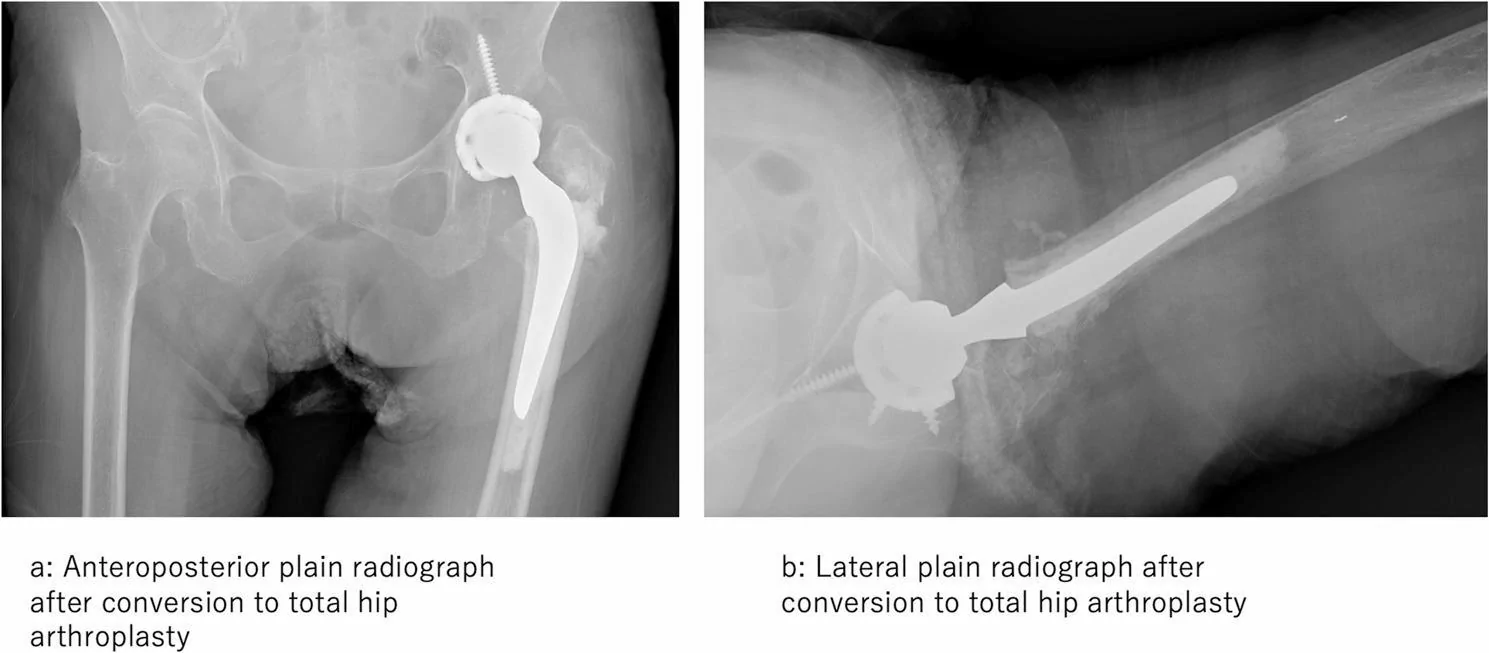
Postoperative imaging after conversion to total hip arthroplasty. **a **Anteroposterior plain radiograph after conversion to total hip arthroplasty via the AMIS approach, performed 1 week after the failed closed reduction. A cemented AMIS-K femoral stem (Size 2S1 Standard) with a 32-mm ceramic head, an Mpact 3D Metal Multi Hole acetabular cup (φ46 mm) with a highly cross-linked polyethylene liner, and Simplex bone cement (Stryker) were used. **b** Lateral plain radiograph after conversion to total hip arthroplasty, confirming acceptable sagittal alignment of the femoral stem. The greater trochanteric fragment remained in stable apposition without supplemental fixation

Because open reduction had already been considered necessary, the intertrochanteric fracture had not united, and early weight-bearing and restoration of offset were considered essential, conversion to total hip arthroplasty was selected as the salvage procedure. Total hip arthroplasty was performed 1 week after the failed closed reduction using the AMIS-K anterior approach. The primary goal of salvage surgery was to prevent redislocation. During arthroplasty, the longest possible neck was selected and offset and leg length were restored as much as possible. Stability was confirmed intraoperatively by ensuring that the hip did not dislocate during flexion and external rotation. The acetabular component was an Mpact 3D Metal Multi Hole cup (φ46 mm; Medacta International, Castel San Pietro, Switzerland) with a highly cross-linked polyethylene liner, and the femoral component was an AMIS-K stem (Size 2S1 Standard; Medacta International) with a 32-mm ceramic head (MECTACERA Biolox Delta). Cementation was performed with Simplex bone cement (Stryker) (Fig. 5).

At 1 year after total hip arthroplasty, no redislocation or other complication had occurred. Although the patient had not regained independent ambulation, no further instability event was observed.

### Ethical considerations

The study protocol was approved by the institutional review board of the authors’ institution and was conducted in accordance with the Declaration of Helsinki. Because of the retrospective nature of the study, the requirement for written informed consent was waived by the institutional review board, and patients were informed of the study through an opt-out process.

## Discussion

The first notable feature of this case is its rarity. Postoperative anterior hip dislocation after fracture fixation is uncommon, and the previously reported cases have mainly involved screw-plate fixation for trochanteric fractures [3–5]. To our knowledge, this is the first reported case of atraumatic anterior hip dislocation after cephalomedullary nailing for an intertrochanteric femoral fracture. Nevertheless, the principal lesson of this case extends beyond its rarity: it illustrates how a combination of fracture-specific biomechanical factors, suboptimal reduction strategy, and a necessary intraoperative soft-tissue maneuver may converge to produce an atypical and serious complication.

The reverse oblique fracture pattern (AO/OTA 31-A3) presents particular technical challenges that are directly relevant to the present case. In this fracture configuration, the lesser trochanter remains attached to the distal fragment rather than the proximal fragment. As a result, the proximal fragment is deformed into abduction and external rotation by the gluteal muscles and short external rotators, whereas the distal fragment is pulled into flexion, external rotation, and adduction by the iliopsoas and adductor muscles. This opposing deformity pattern makes anatomical reduction inherently difficult and, together with the obliquity of the fracture line, hinders the creation of positive medial cortical support [1, 10, 13, 14]. Unlike more stable intertrochanteric patterns, the reverse oblique configuration does not allow reliable bony interlocking at the medial cortex, even when reduction appears acceptable on fluoroscopy. Moreover, because the deforming forces on the proximal and distal fragments act in opposite directions, slight over-reduction toward positive medial cortical contact is technically difficult to maintain. Consequently, the construct may be guided toward progressive shortening and telescoping unless sufficient cortical buttress is created at the index surgery. For this reason, a slight over-reduction targeting positive medial cortical contact—rather than purely anatomical alignment—has been advocated to resist progressive varus collapse and excessive telescoping under load [10]. In the present case, reduction was anatomical, but in retrospect a one-cortex-width positive medial reduction would have been preferable to provide a bony buttress against progressive shortening. The absence of adequate cortical interlocking meant that no secondary mechanical resistance was available once telescoping began.

Several mechanical factors likely contributed to anterior instability in a cumulative fashion. Progressive telescoping of 8.96 mm, which meets the definition of excessive sliding in the cephalomedullary nail literature [11], may have played a central role in the development of instability. The TFNA system incorporates a preassembled locking mechanism intended to limit telescoping of the cephalic element. In the present case, the mechanism was tightened manually to the maximum setting in an effort to minimize postoperative telescoping; however, progressive excessive telescoping was not prevented. In this reverse oblique pattern, the opposing deforming forces acting on the proximal and distal fragments likely not only made positive medial cortical support difficult to achieve, but also promoted progressive shortening once sliding began. In the reverse oblique pattern, the fracture geometry does not generate the cortical contact needed to arrest sliding [10, 13], and once telescoping progresses beyond a critical magnitude, both femoral offset and effective limb length may be reduced. Such loss of offset and length has been shown to compromise the abductor moment arm and reduce periarticular soft-tissue tension around the hip [15], and post-fixation femoral neck shortening has been associated with inferior hip function in clinical series [16]. We hypothesize that this loss of tension may represent the proximate mechanism of anterior instability, although this cannot be directly verified in a single case. Femoral anteversion of approximately 31°, measured using the posterior condylar axis, exceeds the widely accepted normal adult range and approaches the threshold for extreme anteversion [17], which may have further biased the hip toward anterior displacement once soft-tissue tension was compromised. Persistent nonunion and lack of medial cortical healing confirmed by computed tomography indicated that no secondary bony stability had developed to compensate for the mechanical insufficiency of the construct. These factors—excessive telescoping, increased anteversion, and failure of medial cortical healing—are best understood as interacting contributors rather than independent causes.

Intraoperative management of the anterior soft tissues also warrants brief consideration. In reverse oblique intertrochanteric fractures, the fracture line may pass through or adjacent to the attachment zone of the iliofemoral ligament on the intertrochanteric line [13, 18]. In the present case, a small elevator was introduced along the anterior intertrochanteric line to facilitate reduction, which involved limited disruption of the anterior capsule and iliofemoral ligament—the principal static restraint against anterior displacement of the hip [19]. The degree of capsular disruption could not be quantified in this case, and its contribution to instability remains speculative. Nonetheless, given that capsular stripping has been implicated in previously reported cases of post-fixation hip dislocation [3], the possibility that even limited anterior capsular injury contributed to reduced soft-tissue restraint in this mechanically compromised hip cannot be entirely excluded.

The practical implications of this case can be summarized as follows. In reverse oblique intertrochanteric fractures, purely anatomical reduction may be insufficient: a slight positive medial cortical support should be the target to provide a bony buttress against progressive telescoping [10]. Postoperative shortening should be monitored carefully, because in this fracture pattern the absence of cortical interlocking means that excessive telescoping can develop insidiously and may manifest not only as varus collapse or cut-out, but also as an atypical instability pattern such as anterior dislocation [3–5, 10, 11]. Cement augmentation, while useful for enhancing fixation in osteoporotic bone, may not substitute for adequate cortical support, and emerging evidence suggests it can alter the failure mode rather than abolish it [20–22]. Partial weight-bearing or a more cautious loading protocol may also be worth considering in reverse oblique fractures with suboptimal cortical contact, given the known tendency for uncontrolled telescoping in this pattern.

Several considerations underlay the failure of closed reduction and the subsequent decision to proceed with total hip arthroplasty rather than open reduction alone. Under fluoroscopy, no fixed mechanical block from the implant or interposed bone fragments was apparent, and the femoral head resisted reduction despite full muscle relaxation. We interpreted this pattern as most consistent with soft-tissue obstruction—possibly including capsular contracture or peri-articular proliferative tissue around the displaced head—rather than a discrete hard block. The exact timing of dislocation was uncertain, but the clinical course suggested a relatively recent event, likely within approximately one to two weeks. Even this short interval may, however, have been sufficient for adaptive soft-tissue changes that impeded closed reduction.

Open reduction without arthroplasty was considered but ultimately rejected. The principal reasons were the established nonunion of the intertrochanteric fracture and the persistent mechanical instability of the construct: even if open reduction could have successfully relocated the femoral head, the underlying conditions responsible for the dislocation—loss of femoral offset, residual instability at the fracture site, and the inability to restore reliable bony support—would have remained, with a continued risk of redislocation or further failure. Conversion to total hip arthroplasty therefore allowed simultaneous reduction, restoration of offset and leg length, and early weight-bearing, and was considered more appropriate as a salvage procedure in this medically frail patient.

The selection of both surgical approach and femoral component in this conversion arthroplasty warrants explicit consideration, as reverse oblique intertrochanteric fractures commonly involve the greater trochanteric region and may compromise the proximal bone stock available for stem fixation. In the present case, the greater trochanter was a separate but stably positioned fragment without significant displacement, suggesting that the abductor lever arm was largely preserved. The anterior minimally invasive surgical (AMIS) approach [23, 24] was selected for several reasons specific to this case. First, because the failure had manifested as an anterior dislocation, the anterior soft tissues were already compromised; an anterior approach allowed direct visualization and assessment of the anterior capsule and surrounding structures along the same path through which the dislocation had occurred. Second, by avoiding any dissection of the posterior capsule and short external rotators, the posterior soft-tissue restraints—well recognized as important contributors to overall hip stability after arthroplasty—could be entirely preserved, with the aim of minimizing the risk of redislocation [24]. Third, the AMIS approach is muscle-sparing and is intended to facilitate early mobilization, which was particularly important in this elderly, medically frail patient in whom prolonged recumbency carried substantial systemic risks.

A cemented standard-length primary stem (AMIS-K) was chosen over a long revision stem or proximal femoral replacement based on the following considerations. The diaphyseal bone quality was adequate, and a line-to-line cementing technique was expected to provide robust circumferential cement fixation along the proximal-to-distal extent of the stem, allowing axial load to be distributed across the diaphysis rather than being concentrated at the metaphysis. Cement fixation also provided immediate structural stability in this osteoporotic bone, permitting full weight-bearing without reliance on biological ingrowth. Proximal femoral replacement and long revision stems are typically reserved for younger patients or for situations with extensive proximal bone loss or diaphyseal compromise; in this patient with a contained proximal defect and intact diaphysis, these options were considered to carry disproportionate surgical morbidity without a clear mechanical advantage. Supplemental trochanteric fixation with cerclage wiring or cabling was not performed because the greater trochanteric fragment remained in stable apposition and the abductor mechanism was clinically intact at the time of arthroplasty. We acknowledge that the long-term durability of a standard cemented stem in the presence of residual trochanteric discontinuity has not been definitively established, and ongoing follow-up will be necessary. The absence of redislocation, implant subsidence, or trochanteric migration at one-year follow-up provides early reassurance that the chosen strategy was appropriate for this patient.

The role of cement augmentation in this case deserves further consideration. In osteoporotic bone, cement augmentation through a perforated helical blade is intended to enhance fixation purchase and reduce the well-recognized risks of cut-out and cut-through [20]. In the present patient, the neck-shaft angle of 130° was largely preserved from the immediate postoperative period through the time of dislocation despite progressive telescoping of 8.96 mm, suggesting that classical intra-osseous failure modes—including substantial varus collapse, cut-out, and cut-through—were largely averted. Cement augmentation around the blade, however, does not abolish the controlled axial sliding of the cephalic element along the nail axis, nor does it stabilize the distal fragment relative to the nail. Finite element analysis has suggested that cement augmentation may redistribute peak stresses away from the femoral head toward the blade–nail junction and femoral shaft, potentially altering rather than eliminating mechanical demands on the construct [21]. A recent case report similarly described a cement-augmented construct in which the typical cut-out pattern was prevented but failure subsequently occurred through an alternative mechanism, supporting the view that augmentation may not neutralize stresses arising from suboptimal positioning or reduction [22]. We therefore tentatively speculate that, in this reverse oblique fracture with marginal medial cortical support, cement augmentation may have contributed to shifting the predominant failure mode away from intra-osseous failure and toward an atypical peri-articular instability pattern. This hypothesis is consistent with, but cannot be proven by, the present single case.

Joint laxity was not considered a major causative factor in this case. The Beighton score was 1/9, although it may have been underestimated because of age-related osteoarthritis and contractures. Assessment of ligamentous laxity may nonetheless be worthwhile when evaluating postoperative hip instability, as soft-tissue laxity could contribute to insufficiency of the anterior restraints. In the present patient, however, the mechanical factors described above were considered of greater clinical relevance than constitutional laxity.

Several limitations should be acknowledged. The exact timing of dislocation was not documented, the limb position at the time of dislocation was unknown, and spinopelvic factors and combined anteversion of the femur and acetabulum were not fully assessed. The extent of anterior capsular disruption during the index procedure was not directly visualized intraoperatively or postoperatively, and its actual contribution to anterior instability remains speculative. The degree to which the index reduction fell short of optimal medial cortical support cannot be quantified retrospectively with certainty. Finally, as a single-case observation, the proposed mechanisms must be regarded as hypotheses that cannot be proven from this report alone and require corroboration from additional cases or biomechanical studies.

## Conclusion

Atraumatic anterior hip dislocation after cephalomedullary nailing for an intertrochanteric femoral fracture is extremely rare, and to our knowledge has not been previously reported. The principal value of this case lies less in its rarity than in the practical lessons it offers for the management of reverse oblique intertrochanteric fractures. We suggest that surgeons consider targeting positive medial cortical support rather than purely anatomical reduction, monitor postoperative shortening carefully, and remain aware that anterior capsular dissection—although sometimes anatomically required for reduction—may reduce soft-tissue restraint in an already mechanically vulnerable hip. In the era of cement-augmented fixation, recognition that augmentation can alter rather than abolish failure modes is also important, as instability may emerge in place of more familiar complications such as cut-out or nonunion.

## Data Availability

The datasets used and/or analysed during the current study are available from the corresponding author on reasonable request.
